# Supplementary material for: CoPPIs algorithm: a tool to unravel protein cooperative strategies in pathophysiological conditions
Source: Brief Bioinform. 2025 Apr 7;26(2):bbaf146. doi: 10.1093/bib/bbaf146 (PMC11975363; doi:10.1093/bib/bbaf146)
Supplement: Supplementary_Information_and_Figures_rev_bbaf146 [file supplementary_information_and_figures_rev_bbaf146.pdf]

ORIGINAL ARTICLE

Journal Section

CoPPIs Algorithm: A Tool to Unravel Protein Cooperative Strategies in Pathophysiological Conditions

Andrea Lomagno[1] | Ishak Yusuf[1] | Gabriele Tosadori[2] | Dario Bonanomi[3] | Pierluigi Mauri[1,4] | Dario Di Silvestre[1]

[1]Clinical Proteomics Laboratory, Elixir Infrastructure, Institute for Biomedical Technologies - National Research Council, F.lli Cervi 93, 20054 Segrate, Milan, Italy.

[2]Institute of Microbiology, Czech Academy of Sciences, Vídeňská 1083, 14200 Praha 4, Czech Republic.

[3]Division of Neuroscience, IRCCS San Raffaele Scientific Institute, Olgettina 60, 20132 Milan, Italy.

[4]Institute of Experimental Endocrinology and Oncology "G. Salvatore" - National Research Council, Pietro Castellino 111, 80131 Naples, Italy.

Correspondence
Dario Di Silvestre, Clinical Proteomics Laboratory, Elixir Infrastructure, Institute for Biomedical Technologies - National Research Council, F.lli Cervi 93, 20054 Segrate, Milan, Italy.
Email: dario.disilvestre@itb.cnr.it

Funding information

KEYWORDS
Proteomics, Network models, PPI, Co-Expression, Network Topology

1 | SUPPLEMENTARY INFORMATION

1.1 | Transformation of non-significant correlations

Objective: Normalize non-significant p-values and remove values equal to 1, to make logarithmic transformation possible. The transformation is applied just to non-significant correlations to assign a penalty proportional to the uncertainty of correlation values.

The range of p-values from

$$\left(\alpha; 1\right] \tag{1}$$

to a weight coefficient range

$$\left(0; 1\right) \tag{2}$$

where α=0.05
The 1 is excluded from the interval because $log\left(1\right)$ = 0 and we are following the hypothesis that, if there is an interaction, there is a correlation.

1. Calculation of the New Maximum for P-values

$$\text{new\_max} = \begin{cases} \frac{1+\max\left(\text{p.adj}\setminus\{1\}\right)}{2}, & \text{if } \max\left(\text{p.adj}\right) = 1 \\ \max\left(\text{p.adj}\right), & \text{otherwise} \end{cases}$$

The new maximum is equal to the maximum of p-values If the maximum of p-values is equal to 1, the average between 1 and the second maximum is considered as new maximum.

2. Calculation of ϵ and k normalization parameters
The parameters ϵ and k are used in the formula

$$\text{p.adj\_norm1} = \left(\text{p.adj} + \epsilon\right) \cdot k \tag{3}$$

and they are calculated by solving the following system of equations:

$$\begin{cases} \alpha = \left(\alpha + \epsilon\right) \cdot k \\ \text{new\_max} = \left(\max\left(\text{p.adj}\right) + \epsilon\right) \cdot k \end{cases}$$

where

$$\epsilon = \frac{\left(\text{new\_max} - \max\left(\text{p.adj}\right)\right) \cdot \alpha}{\alpha - \text{new\_max}}$$

and

$$k = \frac{\text{new\_max} \cdot \alpha - \max\left(\text{p.adj}\right)}{\alpha \cdot \text{new\_max} - \max^2\left(\text{p.adj}\right)}$$

3. Applying Normalization
   Normalization is applied only to non-significant p-values $\left(\text{p.adj} > \alpha\right)$:

$$\text{p.adj\_norm1} = \left(\text{p.adj}_{\left(>\alpha\right)} + \epsilon\right) \cdot k \tag{4}$$

4. Negative logarithm is set to invert low p-values into high weights and vice versa:

$$\text{lg} = -\log_{10}\left(\text{p.adj\_norm1}\right)$$

5. Normalization of lg between 0 and 1:
   The parameters $\epsilon_{\log}$ and $k_{\log}$ are calculated by solving the following system of equations:

$$\begin{cases} \left(\text{max\_lg} + \epsilon_{\log}\right) \cdot k_{\log} = 1 \\ \left(\text{min\_lg} + \epsilon_{\log}\right) \cdot k_{\log} = \text{min\_lg} \end{cases}$$

   where

$$\text{max\_lg} = -\log_{10}\left(\alpha\right)$$

$$\epsilon_{\log} = \frac{\text{min\_lg} - \text{max\_lg} \cdot \text{min\_lg}}{\text{min\_lg} - 1}$$

   and

$$k_{\log} = \frac{\text{new\_max} \cdot \alpha - \max(\text{p.adj})}{\alpha \cdot \text{new\_max} - \max(\text{p.adj})^2}$$

6. Apply normalization:

$$\text{p.adj\_norm2} = \left(\text{lg} + \epsilon_{\log}\right) \cdot k_{\log}$$

7. Calculating the Final Weights
   The correlation's score is multiplied by p.adj_norm2[3]:

$$\text{weights} = \begin{cases} \text{cor}, & \text{if p.adj} \leq \alpha \\ \text{cor} \times \text{p.adj\_norm2}^3, & \text{if p.adj} > \alpha \end{cases}$$

Raising the adjusted p-value to the third power allows us to exponentially emphasize deviations from the significance threshold. This approach results in weights that are consistent with the *Spearman*'s correlation when p-values are near the significance threshold, while weights tend toward zero when p-values are further from significance.

1.1.1 | CoPPIs score

To assess a different status of correlation of a given functional module in a pair of groups/conditions, CoPPIs computes the ratio K and standard deviations $Sd\left[N_i\right]$, where:

$$K = \frac{Av.TotalCorrG1}{Av.TotalCorrG2} \tag{5}$$

$$Sd_{G1} = \frac{\frac{Sd_{\text{AllCorrG1}}}{\sqrt{Nedges_i}}}{2} \quad \text{and} \quad Sd_{G2} = \frac{\frac{Sd_{\text{AllCorrG2}}}{\sqrt{Nedges_i}}}{2} \tag{6}$$

$$SdN_i = \sqrt{Sd_{G1}{}^2 + Sd_{G2}{}^2} \tag{7}$$

If the value of K exceeds 1, we set it to 1, and simultaneously, we adjust the standard deviation as:

$$SdN_{i\,new} = SdN_{i\,old} - \left(K - 1\right) \tag{8}$$

This adjustment is implemented to prevent the selection of processes that, while statistically significant, may exhibit a lower correlation than that observed in the comparison group.

Plotting the k value of all functional modules against the number of edges, considerable dispersion is evident especially with a lower number of edges (**Figure2B**); a progressively decreasing dispersion as the number of edges increases. By Student's *t*-test, CoPPIs checks if the ratio k_i of a given functional module is lower than the normal distribution of ratios from random functional modules with equal number of edges, with mean K and standard deviations SdN_i; thus, it provides the statistical significance of the correlation difference in a pair of compared groups/conditions.

To further evaluate the significance of differentially correlated functional modules, an empirical scoring system for considering both the direction and magnitude of correlation changes across conditions was introduced. The scoring function can be summarized as:

$$CoPPIs\_Score = C \cdot \sqrt{Zscore} \tag{9}$$

where:

$$Zscore = \frac{K - V}{-\log_{10}\left(SdN_i\right)} \tag{10}$$

The CoPPIs_Score is formulated in two different ways based on the cases we are facing. Specifically:

$$R = \frac{N_{\text{edges}} weighted}{N_{\text{edges}} total} = \frac{x}{y} \tag{11}$$

if R$\leq$ 0.5

$$C = \ln\Big[\frac{\left(1 - R^2\right) \cdot x^{\frac{5}{2}}}{\sqrt{R^3}}\Big] \tag{12}$$

while, if R>0.5

$$C = \ln\Big[\frac{\left(1 - 0.5^2\right) \cdot \left(\frac{y}{2}\right)^{\frac{5}{2}}}{\sqrt{0.5^3}}\Big] + \frac{5 \cdot \left(x - \frac{y}{2}\right)}{2x} \tag{13}$$

The first scoring formula (8) takes into account both the ratio R, i.e. the percentage of edges weighted with correlation, and the magnitude of x and y. Thus, with the same percentage-weighted edges (R), it ensures a higher score for functional modules with a higher number of edges; this function is designed to increase as R increases, reaching a maximum before R approaches 0.5. If R>0.5, the scoring formula (9) addresses the challenge presented by the decreasing nature of the formula (7) when R surpasses 0.5. To overcome this issue, R is set equals to 0.5 (9). Furthermore, it includes a derivative-dependent variable component of the R-score of 0.5, multiplied by the difference between the number of observed correlations (x) and half of the total protein-protein interactions (y/2); this block ensures a balanced scoring mechanism that remains sensitive to variations in both the number of weighted edges and the total protein-protein interactions.

Finally, C is multiplied to the square root of the Z score (5). In summary, C is dependent by the percentage of weighted protein-protein interactions, while Zscore is proportional to the number of standard deviations (of random functional modules with an equal number of interactions N

2 | SUPPLEMENTARY FIGURES

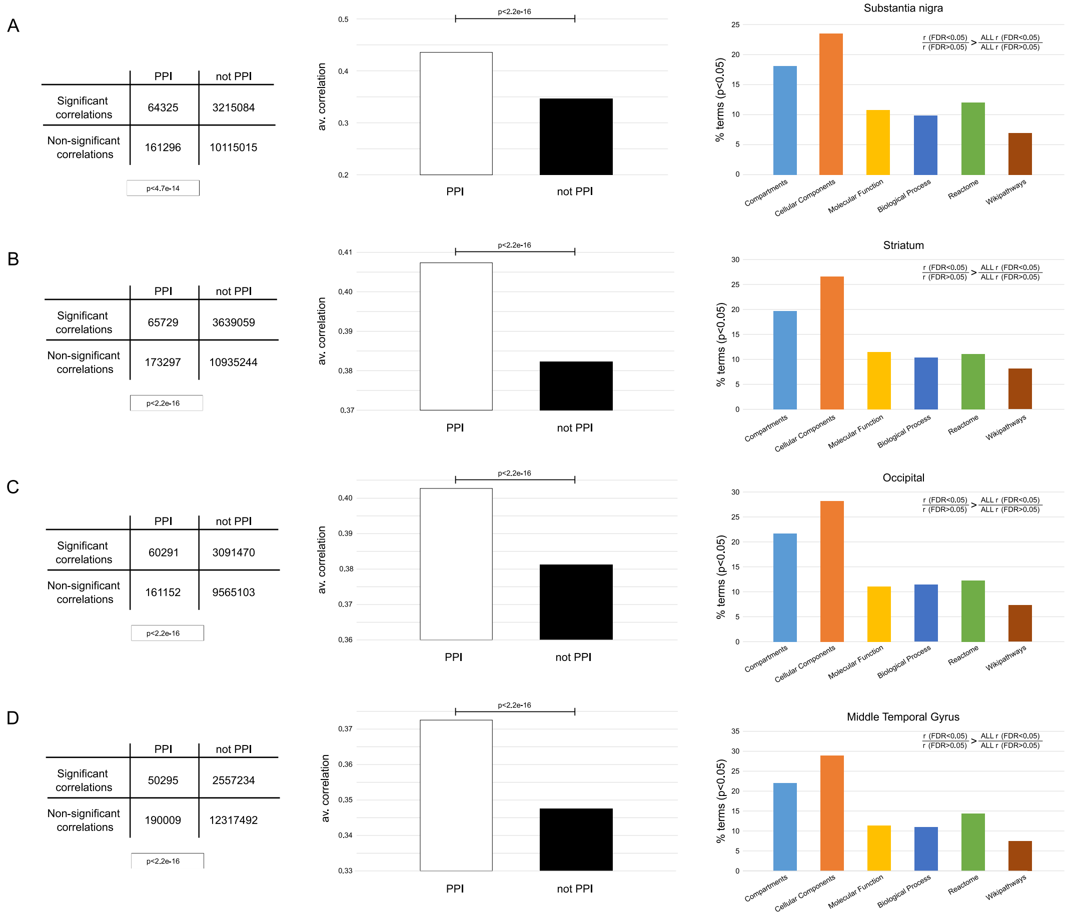

FIGURE 1 PPIs vs not PPIs correlation and percentage of GO terms and pathways with a significant correlation. A) Substantia Nigra, B) Striatum, C) Occipital Cortex, D) Middle Temporal Gyrus From left to right: contingency table showing significant and not significant correlation associated with PPIs and not PPIs; p value from *Fisher*'s test with NULL hypothesis that the ratio is the same. Following, a barplot reporting the average absolute values of significant correlations associate with PPIs and not PPIs; p value from Student's *t*-test with NULL hypothesis that the mean of the distributions are the same. Finally, a barplot reporting the percentage of GO terms and pathways whose corresponding proteins were significantly correlated. They were extracted according to the formula reported in each box, where r is the correlation of a given GO term/Pathway and ALL represents the total average correlations measured. In other words, we applied a *Fisher*'s test with NULL hypothesis that the percentage of significant correlations of each term, over the total correlations, is less or equal than the ratio of all significant correlations over all correlations (FDR$\leq$ 0.05).

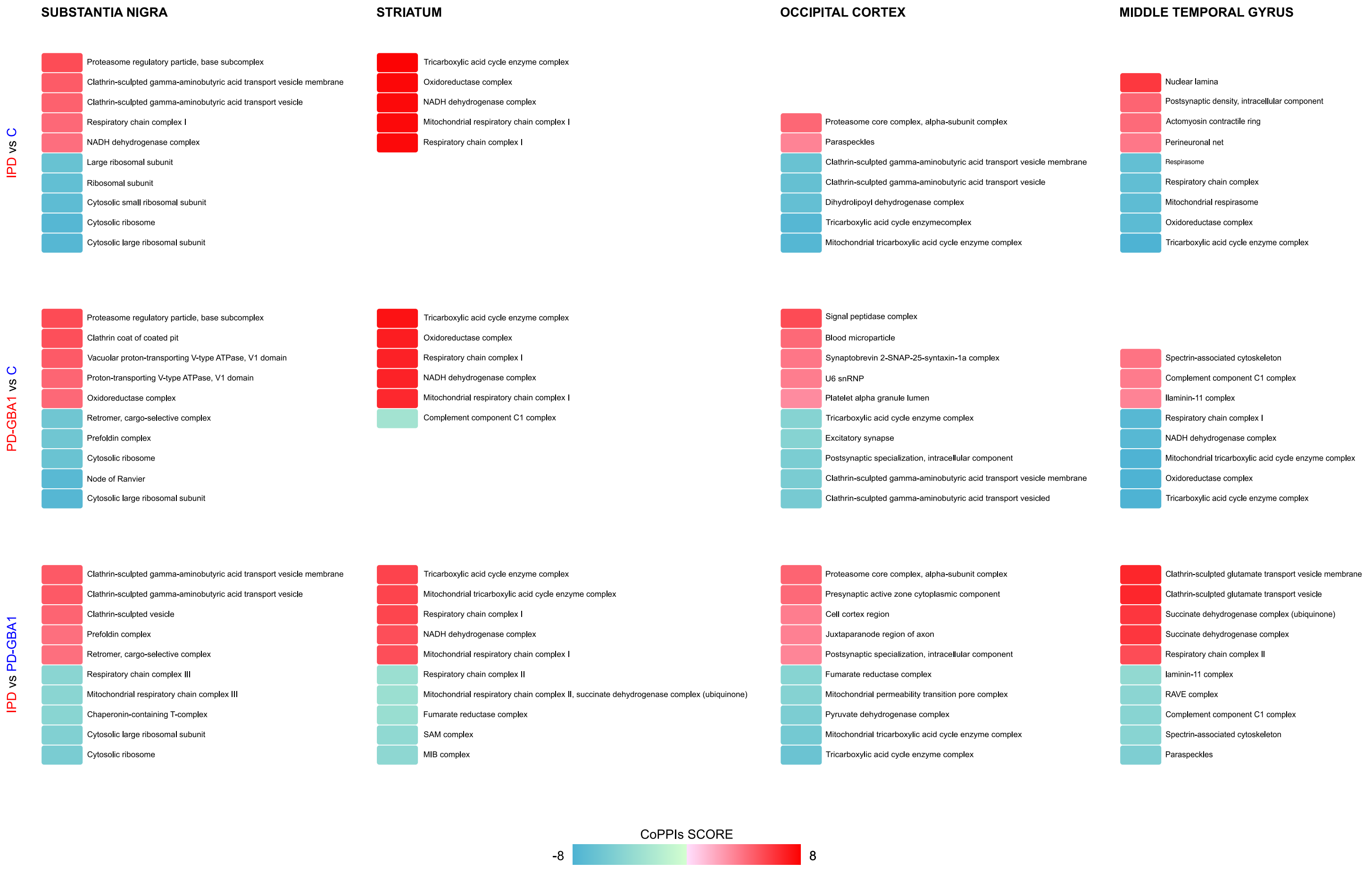

FIGURE 2 Cellular Components (CCs) differentially correlated by comparing the same brain zone (SN, STR, OCC or MTG) of Control, IPD and PD-GBA1 subjects. For each pairwise comparison, the best 5 ranked CCs per condition, based on CoPPIs score, are shown. Specifically, CCs were selected by applying the following thresholds: CoPPIs score $\geq |2|$, percentage of significative edges $\geq 30\%$, p.adj≤ 0.001.

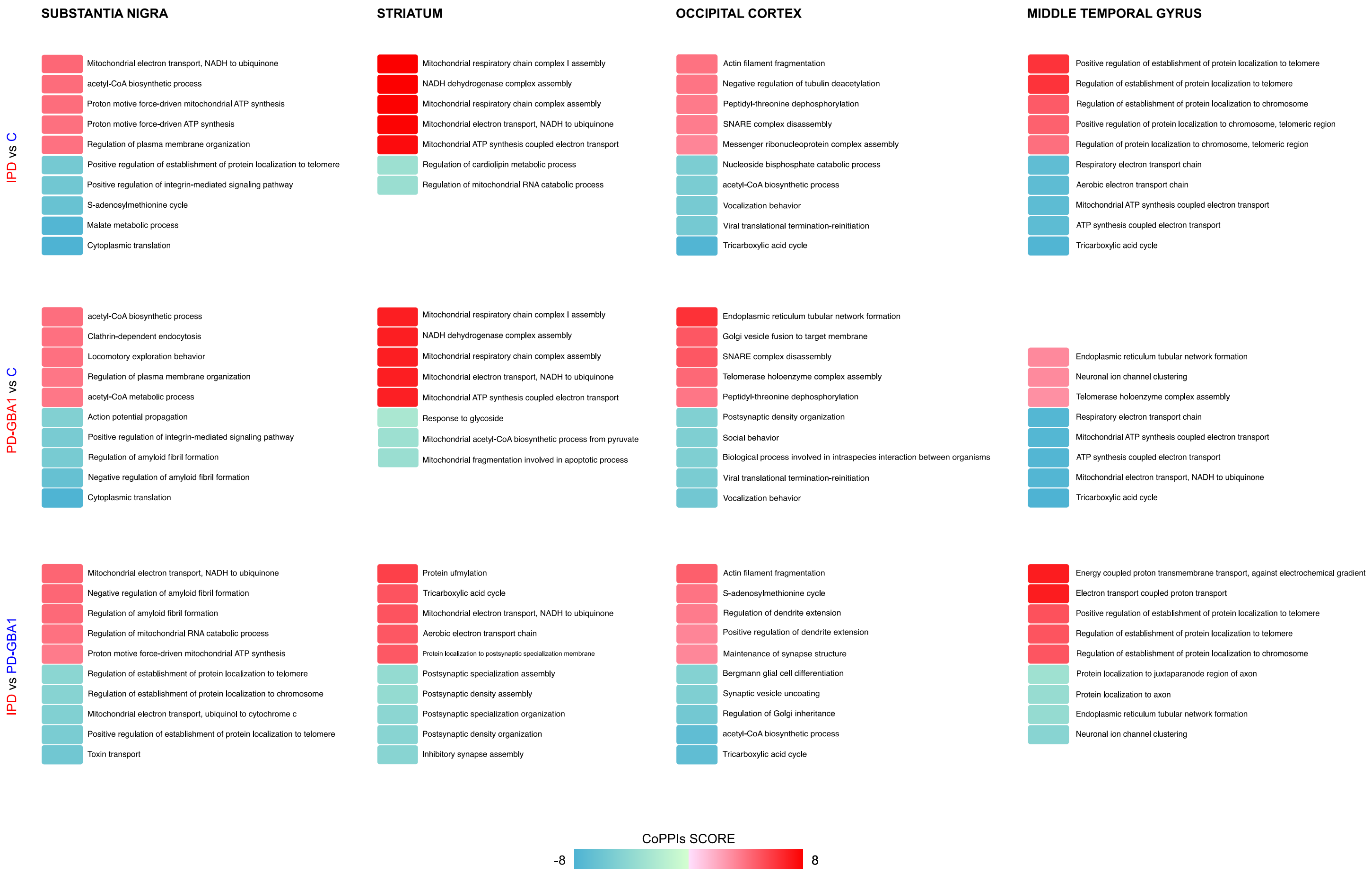

FIGURE 3 Biological Processes (BPs) differentially correlated by comparing the same brain zone (SN, STR, OCC or MTG) of Control, IPD and PD-GBA1 subjects. For each pairwise comparison, the best 5 ranked BPs per condition, based on CoPPIs score, are shown. Specifically, BPs were selected by applying the following thresholds: CoPPIs score $\geq |2|$, percentage of significative edges $\geq 30\%$, p.adj$\leq$ 0.001.

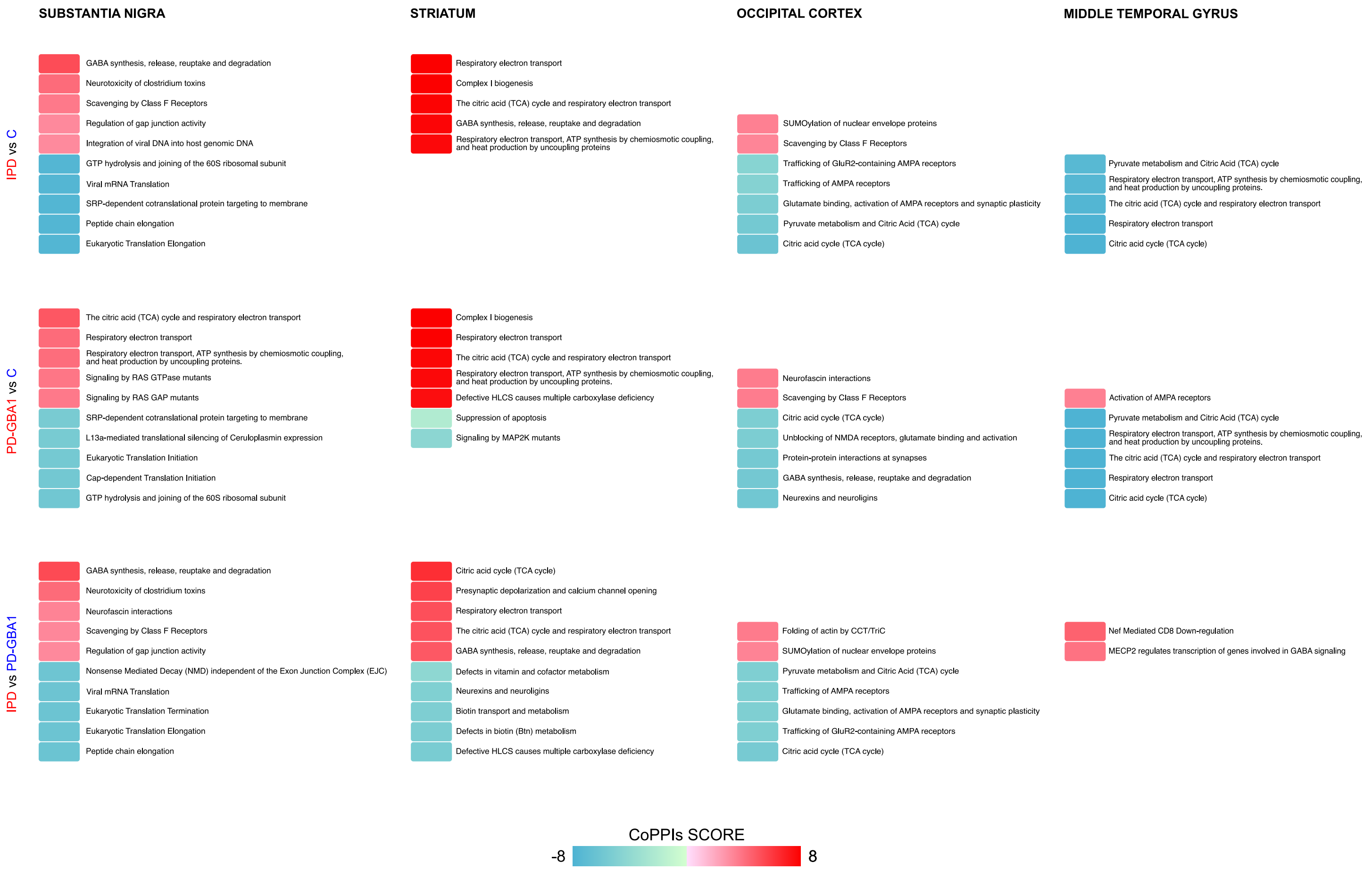

FIGURE 4 REACTOME pathways differentially correlated by comparing the same brain zone (SN, STR, OCC or MTG) of Control, IPD and PD-GBA1 subjects. For each pairwise comparison, the best 5 ranked pathways per condition, based on CoPPIs score, are shown. Specifically, pathways were selected by applying the following thresholds: CoPPIs score $\geq |2|$, percentage of significative edges $\geq 30\%$, p.adj≤ 0.001.

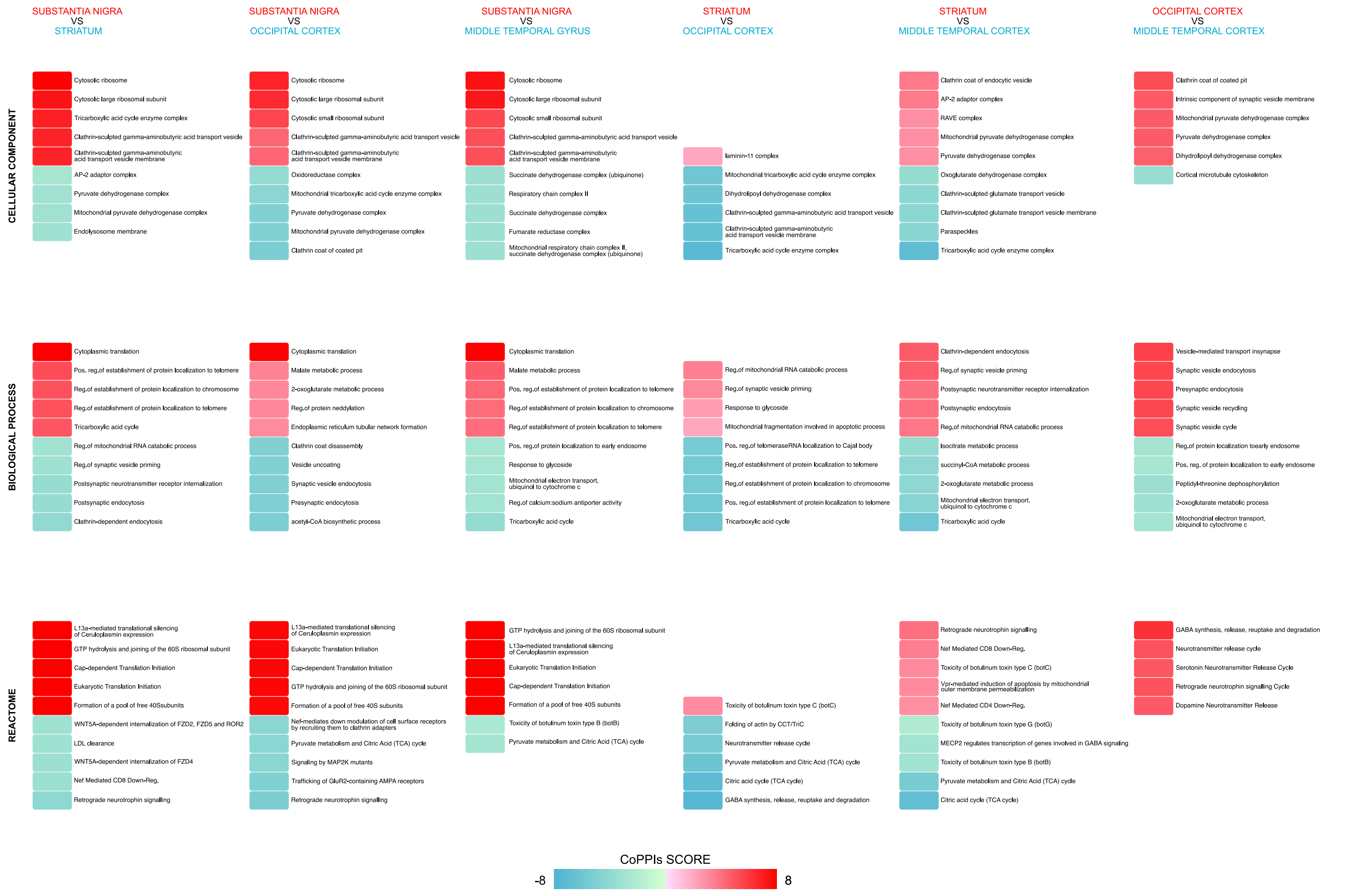

FIGURE 5 Cellular Components (CCs) differentially correlated by comparing different brain zones (SN, STR, OCC and MTG) within Control, IPD and PD-GBA1 groups. For each pairwise comparison, the best 5 ranked CCs per condition, based on CoPPIs score, are shown. Specifically, CCs were selected by applying the following thresholds: CoPPIs score $\geq |2|$, percentage of significative edges $\geq 30\%$, p.adj≤ 0.001.

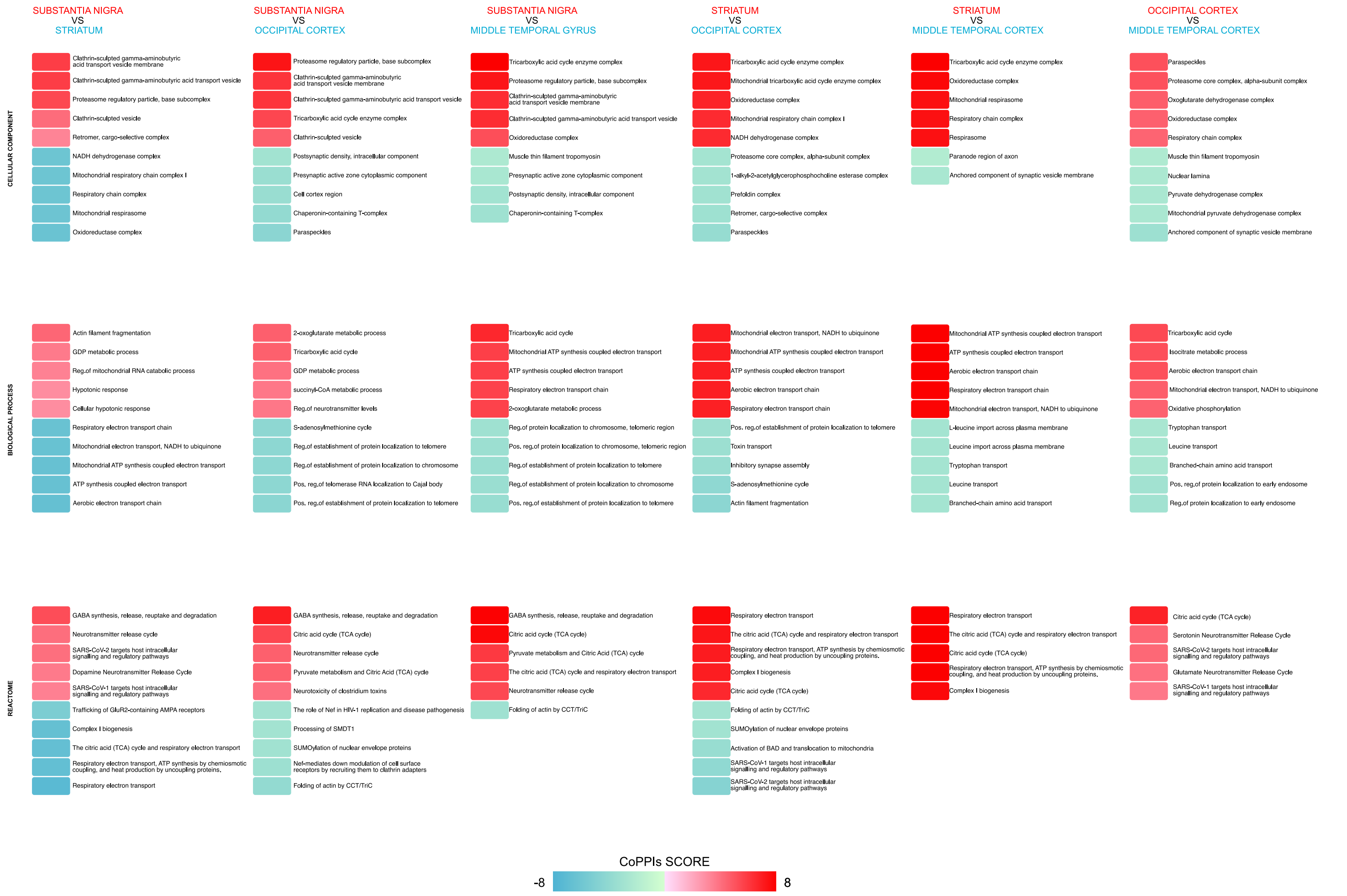

FIGURE 6 Biological Processes (BPs) differentially correlated by comparing different brain zones (SN, STR, OCC and MTG) within Control, IPD and PD-GBA1 groups. For each pairwise comparison, the best 5 ranked BPs per condition, based on CoPPIs score, are shown. Specifically, BPs were selected by applying the following thresholds: CoPPIs score $\geq |2|$, percentage of significative edges $\geq 30\%$, p.adj≤ 0.001.

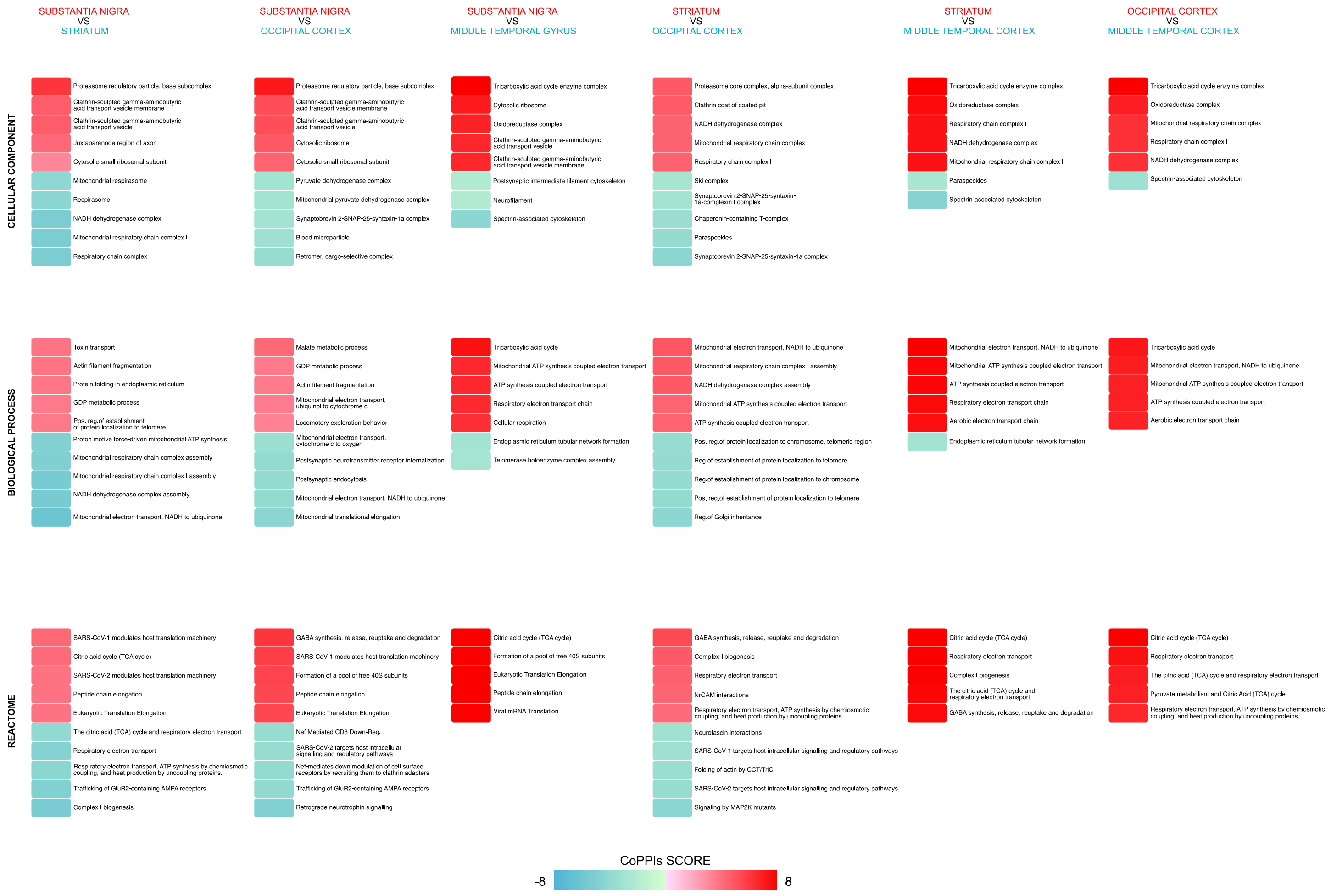

FIGURE 7 REACTOME pathways differentially correlated by comparing different brain zones (SN, STR, OCC and MTG) within Control, IPD and PD-GBA1 groups. For each pairwise comparison, the best 5 ranked pathways per condition, based on CoPPIs score, are shown. Specifically, pathways were selected by applying the following thresholds: CoPPIs score $\geq |2|$, percentage of significative edges $\geq 30\%$, p.adj$\leq$ 0.001.

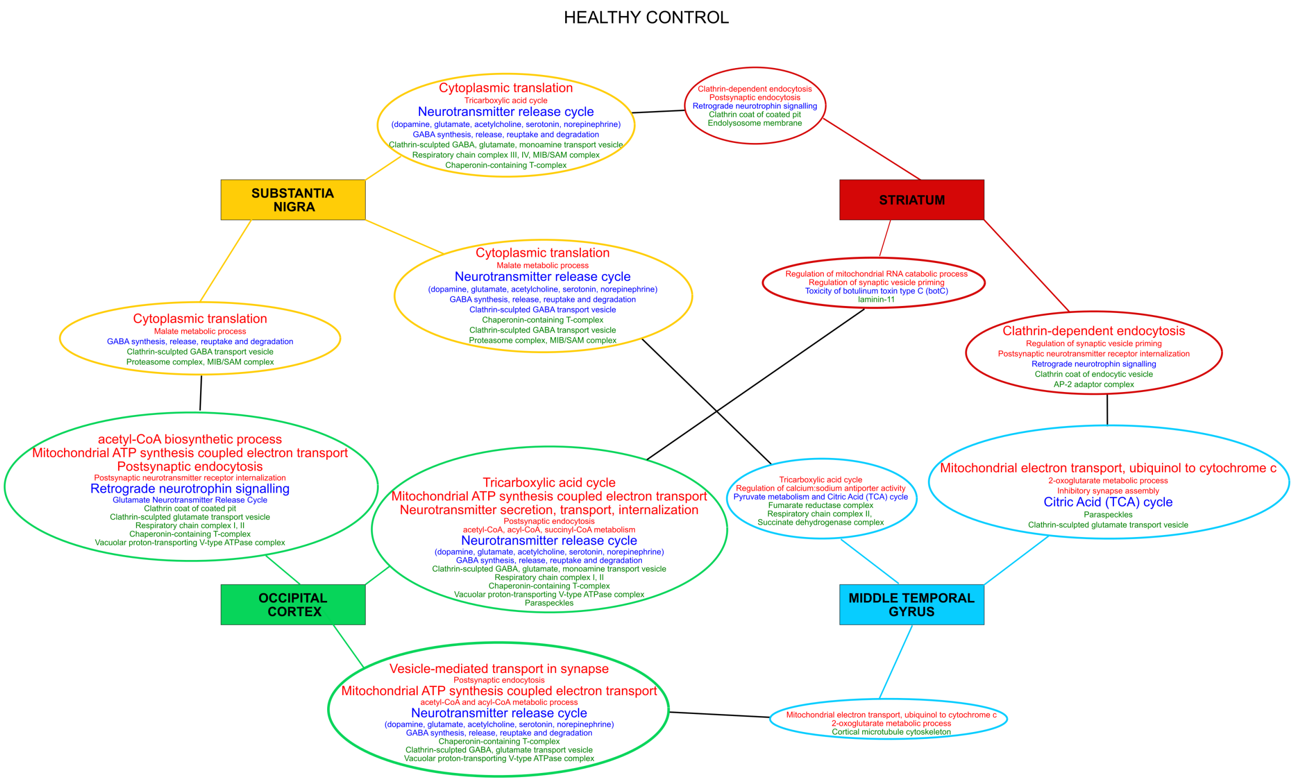

FIGURE 8 Biological processes (red), REACTOME pathways (blue) and Cellular Components (green) differentially correlated by comparing different brain zones within Control groups. Yellow, red, green and blue light circles indicate functional modules more correlated in SN, STR, OCC and MTG, respectively; connected circles refer to pairwise comparison (i.e. yellow-red circles indicate SN vs STR).

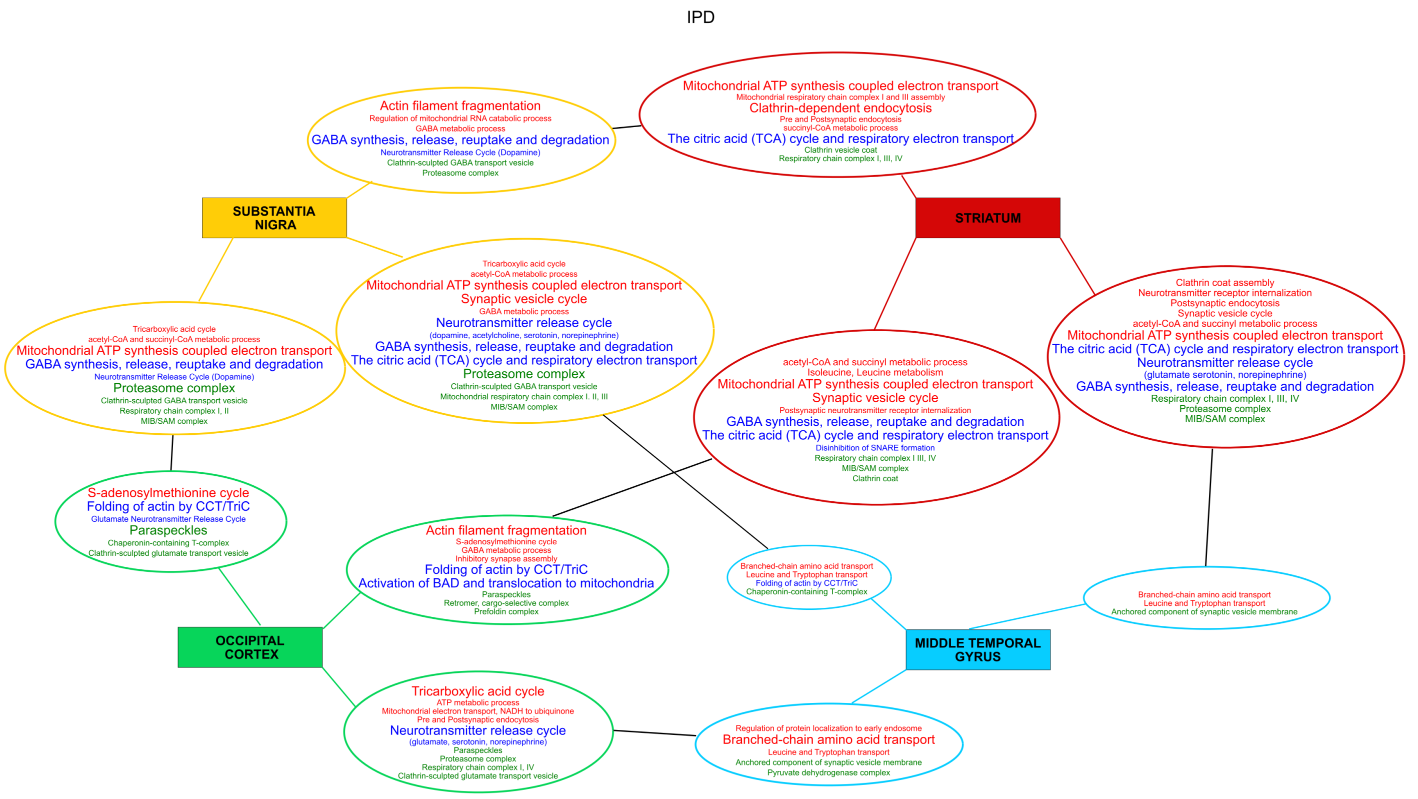

FIGURE 9 Biological processes (red), REACTOME pathways (blue) and Cellular Components (green) differentially correlated by comparing different brain zones within IPD groups. Yellow, red, green and blue light circles indicate functional modules more correlated in SN, STR, OCC and MTG, respectively; connected circles refer to pairwise comparison (i.e. yellow-red circles indicate SN vs STR).

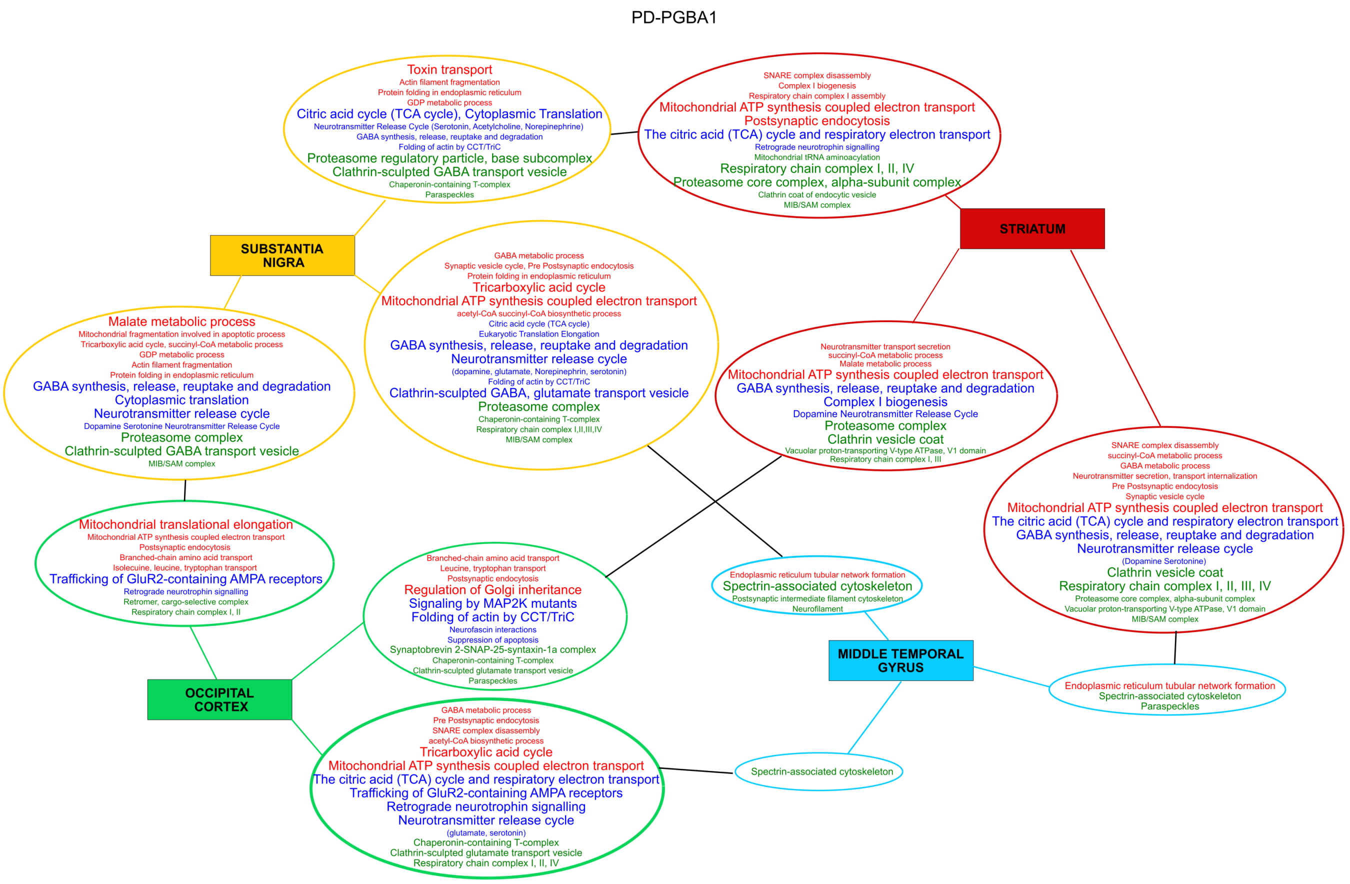

FIGURE 10 Biological processes (red), REACTOME pathways (blue) and Cellular Components (green) differentially correlated by comparing different brain zones within PD-GBA1 groups. Yellow, red, green and blue light circles indicate functional modules more correlated in SN, STR, OCC and MTG, respectively; connected circles refer to pairwise comparison (i.e. yellow-red circles indicate SN vs STR).

A

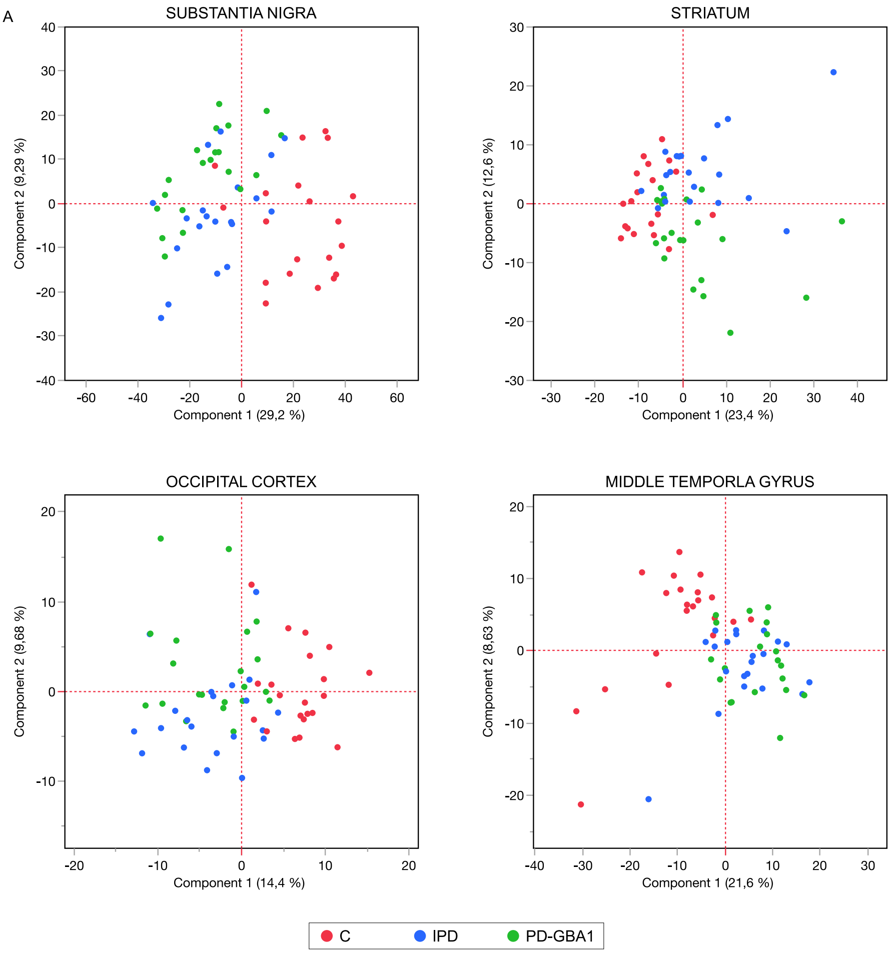

B

SUBSTANTIA NIGRA			STRIATUM			OCCIPITAL CORTEX			MIDDLE TEMPORLA GYRUS		
C vs IPD	C vs PD-GBA1	IPD vs PD-GBA1	C vs IPD	C vs PD-GBA1	IPD vs PD-GBA1	C vs IPD	C vs PD-GBA1	IPD vs PD-GBA1	C vs IPD	C vs PD-GBA1	IPD vs PD-GBA1
930	964	135	294	248	162	179	136	48	252	324	69
510	707	214	310	231	148	159	167	98	260	320	95

FIGURE 11 Label-free quantitative analysis. A) Principal Component Analysis (PCA) starting from differentially expressed proteins (DEPs) in Substantia Nigra, Striatum, Occipital cortex, and Middle Temporal Gyrus. B) DEPs were selected by Linear Discriminant Analysis (LDA, $P<0.05$) by comparing Controls (C), idiopathic Parkinson's disease (IPD) and Parkinson's disease characterized by GBA1 mutations (PD-GBA1). For each comparison, i.e. C vs IPD, the number in red indicates DEPs up-regulated in C, while the number in blue indicates DEPs up-regulated in IPD.

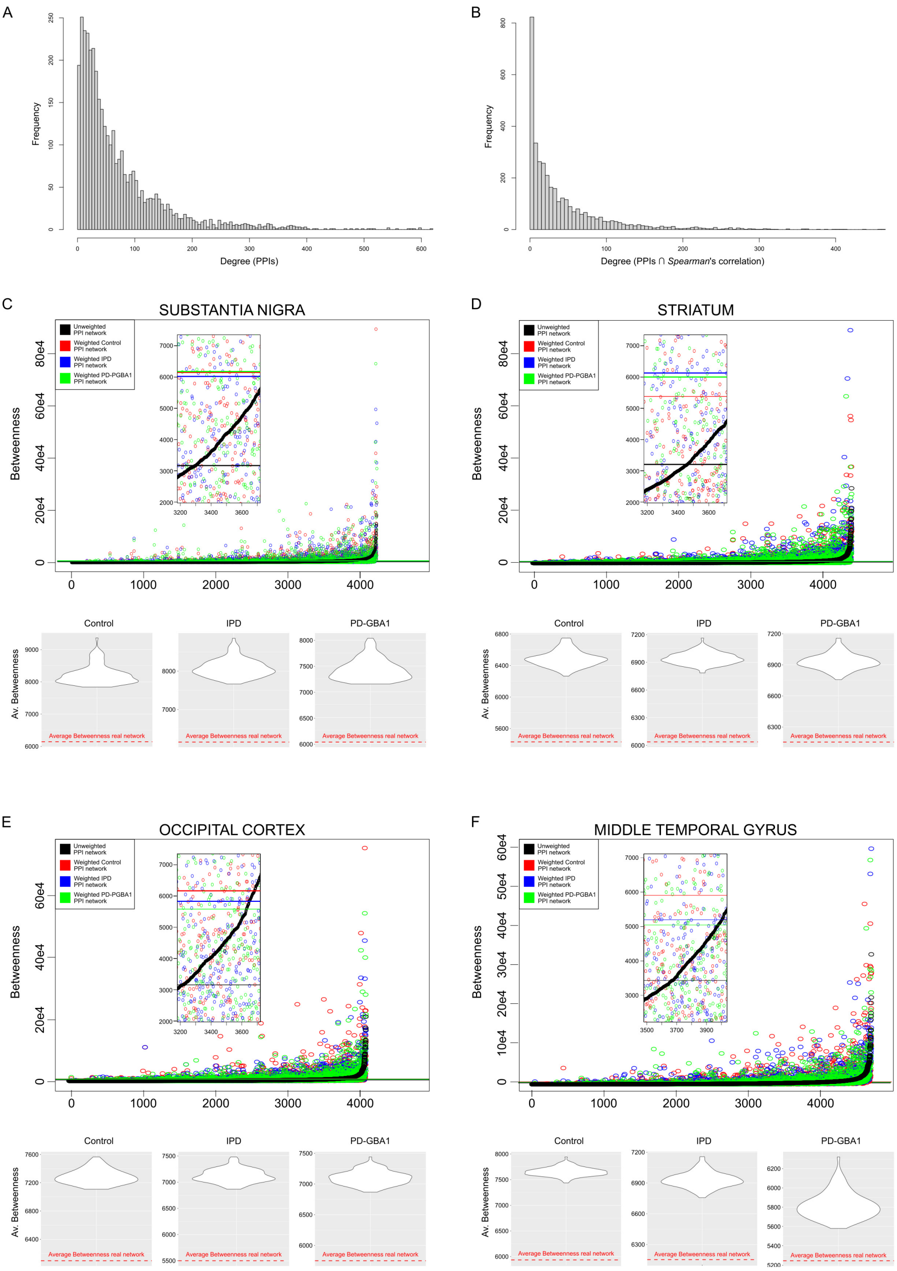

FIGURE 12 Weighted network topological analysis. Degree distribution in A) unweighted and B) weighted PPI networks highlighted scale-free models. The models were reconstructed using proteins found in all brain areas. Weighted PPI model was obtained by considering *Spearman*'s correlation scores $\geq$0.64 and FDR$\leq$0.05; PPIs associated with not significant correlation were removed. C-F) For each brain area, the upper plots show nodes ordered by increasing betweenness in the corresponding unweighted and weighted PPI networks. Nodes from the Control, IPD, and PD-GBA1 networks are represented in red, blue, and green, respectively. Below, violin plots illustrate the distribution of betweenness in random networks with the same degree distribution. The average betweenness of the original network is highlighted in red. It is evident that the mean betweenness consistently differs from the random distribution, indicating a shift away from random behaviour.

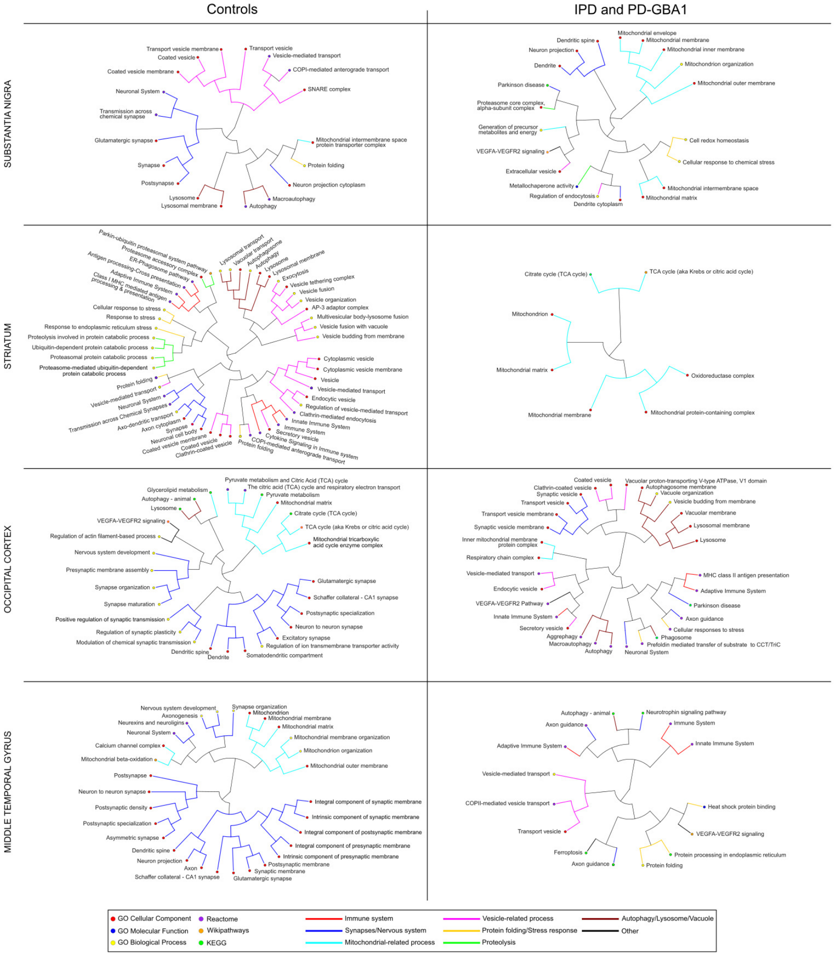

FIGURE 13 Functional analysis of condition- and brain area-specific nodes and bottlenecks. Enriched Cellular Components, Molecular Functions, Biological Processes and Pathways (Reactome, Wikipathway, Kegg) were extracted (FDR$\leq 0.05 and selected by LDAP \leq$ 0.05

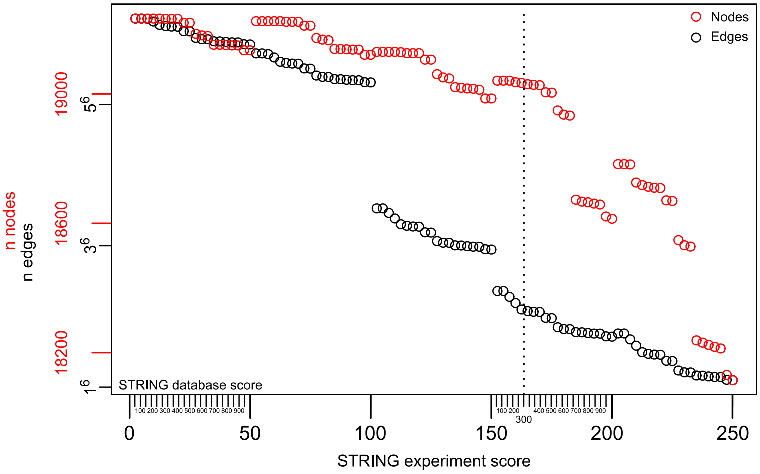

FIGURE 14 This graph represents the combined trend of the number of nodes (in red) and edges (n black) of the human interactome graph, as a function of the thresholds for the STRING "experiment" and "database" scores (x-axis). The thresholds in this graph are multiplied by 1000. We tested all combinations of score thresholds in 50-point increments for both types of scores. After each increment (0, 50, 100, 150, 200) of the 'experiment' score, the smaller numbers (ranging from 100 to 900) indicate the 'database' score thresholds. Considering the 'experiment' score, we observe a drop in the number of edges from 50 to 100 and from 100 to 150, followed by a relatively continuous trend. For this reason, we selected a STRING 'experiment' score threshold of 0.15 (150). In addition, by applying the STRING 'database' score thresholds, we notice a continuous drop in the number of edges until 0.3 (300), after which the trend remains fairly constant. We chose these thresholds to achieve a balance between confidence and a high number of interactions. The use of such thresholds has a double meaning. On the one hand, we aimed to ensure a minimum confidence threshold, on the other hand we wanted to use as many interactions as possible that would be further filtered later through correlation values calculated through experimental proteomic data. Although proteomic technologies today allow to obtain increasingly complete profiles, the large coverage of the proteome remains a limitation. Therefore, the use of such thresholds represents a compromise that aims to increase the matching between PPI models and protein co-expression models reconstructed from experimental data. This also means that these thresholds could be further adjusted depending on the organism we are studying, the coverage of the experimental proteome we define and the completeness of the corresponding available interactome.

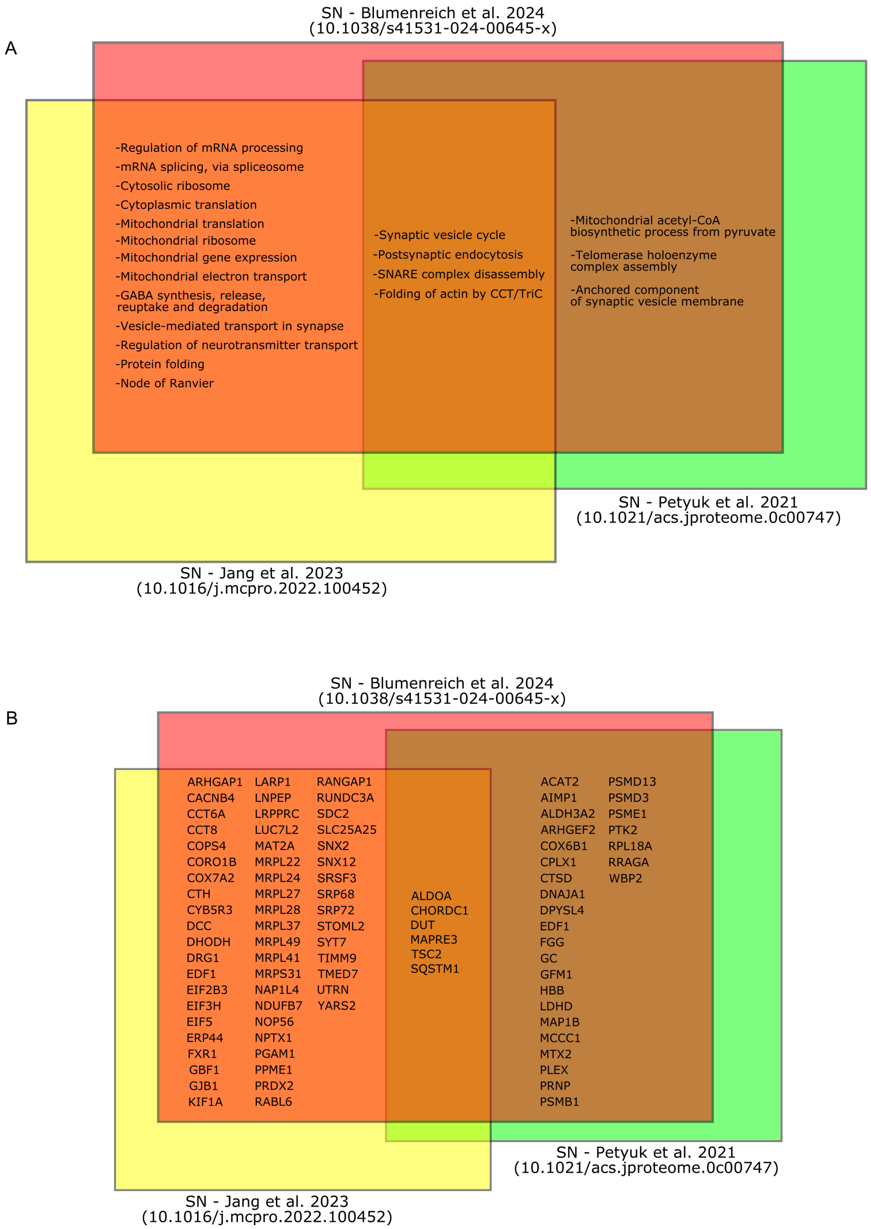

FIGURE 15 Functional modules (A) and hubs/bottlenecks (B) found in common by processing the dataset under study (Blumenreich et al. 2024) and two other independent datasets (Jang et al. 2023 and Petyuk et al. 2021). Due to the limited availability of datasets that meet the features required by the CoPPIs algorithm, only the Substantia Nigra (SN) was considered. The study by Jang et al. 2023 performed an in-depth proteomic analysis of human SN tissues from 15 PD patients and 15 healthy controls, while Petyuk et al. 2021 analyzed Substantia Nigra (SN) tissue from 17 subjects with Lewy body pathology and neuronal loss and 17 controls. Note that a higher number of associations were found between the dataset of our study (Blumenreich et al. 2024) and the cohort of subjects analyzed by Jang et al. 2023. The latter were well classified as patients with Parkinson's disease, while the subjects selected by Petyuk et al., based on Lewy body pathology and neuronal loss, were mostly diagnosed with Alzheimer's disease. This result, combined with the selection of functional modules and hubs/bottlenecks involved in mitochondrial and cytoplasmic translation, further confirm the efficacy of the CoPPIs algorithm and strengthen the involvement of translation in the pathophysiology of Parkinson's disease.
